# Tree Height Prediction Using a Double Hidden-Layer Neural Network and a Mixed-Effects Model

**DOI:** 10.3390/plants15081176

**Published:** 2026-04-10

**Authors:** Jianbo Shen, Xiangdong Lei, Yutang Li, Yuehong Pan, Gongming Wang

**Affiliations:** 1 Wenzhou Key Laboratory of AI Agents for Agriculture, Wenzhou Vocational College of Science and Technology, Wenzhou 325006, China; lyshenjianbo@163.com; 2 Institute of Resources Information Techniques, Chinese Academy of Forestry, Beijing 100091, China; xdlei@caf.ac.cn; 3 Institute of Forestry Inventory and Planning of Jilin Province, Changchun 130022, China; yutanglee@163.com; 4 Agricultural Information Institute, Chinese Academy of Agricultural Sciences, Beijing 100081, China; 5 School of Computer Science and Engineering, Intelligent Collaborative Innovation Studio, Guangzhou Institute of Science and Technology, Guangzhou 510540, China

**Keywords:** tree height prediction, mixed-effects model, double hidden-layer neural network, k-fold cross-validation

## Abstract

The double hidden-layer neural network has increasingly been applied in tree height modeling due to its superior performance. To improve the precision of tree height estimation, this study compared the performance of a double hidden-layer neural network with that of a nonlinear mixed-effects model, aiming to provide a new method for tree height prediction. Taking the *Larix olgensis* forest plantation in Jilin Province as the research object, a double hidden-layer back propagation (BP) neural network was established for tree height prediction by adopting trial and error, k-fold cross-validation, and near-domain optimization strategies. In constructing the nonlinear mixed-effects model, the overall and local differences in forest growth data, as well as the autocorrelation among the various levels of data, were considered. Accordingly, after determining the base model, random effects were introduced, the correlation variance–covariance matrix was calculated, and random parameters were estimated to compare the predictive performance of the two aforementioned models. For the mixed-effects model, the coefficient of determination *R*^2^ was 0.8590, the root mean square error (RMSE) was 1.6230, and the mean absolute error (MAE) was 2.2658. For the double hidden-layer BP neural network, the *R*^2^ reached 0.9068 (an increase of 5.56%), the RMSE was 1.3197 (a decrease of 18.69%), and the MAE was 1.2736 (a decrease of 43.79%). The results demonstrate that the double hidden-layer BP neural network is superior to the nonlinear mixed-effects model for tree height prediction. Therefore, the results provide a more accurate method for tree height prediction.

## 1. Introduction

Tree height is one of the important tree-measuring factors in forest surveys, and it is also an important basis for assessing growth status and site quality as well as dividing the forest layers [1,2]. Since it is difficult to measure directly, tree height is calculated with a model based on the relationship between it and DBH (diameter at breast height). The core of this method is formulating various special equations to obtain the height by solving those equations [3,4,5,6]. Because the stand mean height is affected not only by the DBH, but also by other factors, all factors that affect the tree height should be considered.

In general, the factors affecting tree height include DBH, site factor, forest competition factor, and climate factor [7]. A neural network has numerous characteristics such as nonlinear mapping, adaptability, generalization ability, and fault tolerance. In recent years, it has been gradually applied to forest growth prediction and achieved various results. Scrinzi et al. [8] used neural network models to update the tree DBH distributions for managed alpine stands and predict the number of standing trees. Pirotti [9] analyzed the return signals from canopy covers by using an artificial neural network (ANN) to determine if there is an improvement in detecting tree height and position compared to a more classic local-maximum-filter approach. Diamantopoulou [10] used an ANN model to estimate the inside-bark and outside-bark total volume of dominant pine trees (Pinus brutia) in reforestations. A neural network model is a hot method at present, and some scholars [11,12,13,14,15] have adopted a neural network model to predict forest growth. In the field of forest growth research, the application of neural networks is limited in the single hidden-layer, and the research involving more hidden layers is rarely reported. However, it is considered that double hidden layers are better than a single layer for more complex problems [16]. Therefore, the double hidden-layer neural network is selected to establish a tree height model.

Fixed effects models [17] assume that the observed data are independent and identically distributed. However, in many practical scenarios, such as forest growth modeling, data exhibit hierarchical or spatiotemporal correlation, which violates these assumptions. The mixed-effects models, introduced to address these limitations by incorporating random effects into fixed effects, provide more accurate and robust predictions. It has shown improved performance in handling complex data structures with spatiotemporal correlation, can reflect the overall average change trend, and provide various information reflecting inter-individual differences, such as data variance and covariance. In addition, this method can represent the correlation error by specifying the different covariance structures so as to improve the prediction accuracy and explain the source of random error. Compared with other models, it has advantages in the processing of continuous observed data having spatiotemporal sequence correlation [18]. The tree height data were obtained through several consecutive observations, and a certain autocorrelation exists between the levels of these repeated measurement data. The random error of them consists of the individual random effects and the multiple repeated measurement effects within the individual, for which the mixed model is suitable for dealing. Thus, the mixed-effects model is chosen to predict the tree height. The mixed-effects model can be divided into linear and nonlinear models. Because the tree height is affected by many factors and the interaction effect of them is extremely complicated, it is appropriate to adopt the nonlinear mixed-effects model. Therefore, some nonlinear mixed-effects models for estimation of tree height have been developed in recent years. Razali [19] used mixed-effects models for predicting the early height growth of forest trees planted in Sarawak, Malaysia. Kalbi [18] evaluated two linear and eighteen nonlinear height–diameter equations and took the best model as the base mixed-effects model to predict the tree height of Oriental beech in the Hyrcanian Forest in Iran. Lam [20] utilized the taxonomic hierarchy of genus and species as the random effects of the nonlinear mixed-effects model to develop species-specific height–diameter (H–D) models for 842 species representing 295 genera, which is useful for many rare tree species with very few observations.

Neural networks have been increasingly applied to tree height prediction due to their ability to capture complex nonlinear relationships. However, the performance of neural networks can vary significantly depending on their architecture, such as the number of hidden layers and the choice of activation functions. Thus, this study focuses on comparing the performance of a double hidden-layer neural network with that of a nonlinear mixed-effects model, providing insights into the impact of hidden layer architecture on prediction accuracy. The mixed-effects model introduces random effects into the fixed model, thereby enabling description of both general and individual characteristics, which has high prediction accuracy. However, usage of the complex structure of the mixed-effects model requires strong professional knowledge. In addition, the amount of data required by this method is very large, and the data must have obvious differences. Therefore, the above two methods have advantages and disadvantages. In practical applications, both the neural network and mixed-effects model should be established with the given data, and then they can be compared and analyzed according to the evaluation criteria.

In order to compare the application effect in tree height prediction, the tree height models based on the double hidden-layer back propagation (BP) neural network and the nonlinear mixed-effects model were respectively established with the data of the *Larix olgensis plantation* in Jilin Province, and the fitting accuracy between them was compared. The research aims to provide a reference for tree height model optimization.

## 2. Materials and Methods

### 2.1. Data Sources

In this study, data were obtained from the forest inventory in Jilin Province in 2009, covering a total of 7662 trees across 96 sample plots. To ensure the research subjects were valid and stable forest stands, and to exclude interference from non-target plots such as newly afforested but not yet matured areas and sparse forests, this study excluded plots with a forest stand density below 300 trees per hectare. Additionally, abnormal values in measurements such as tree height and age were removed to eliminate the impact of measurement errors and extreme individuals on model fitting, thereby ensuring the homogeneity and reliability of the data. The final dataset included a total of 7001 trees across 86 sample plots. Although the number of sample plots is not large, the number of trees is over 80 times that of the sample plots. Each record of data represents all the information within the corresponding sample plot. Behind this lies a huge amount of data, which is representative and typical, and the data quality is relatively high. In our experiment, 64 records were taken as the training data and others as the testing data. In this study, the DBH, stand basal area, and slope position (the position of the slope where the plot was surveyed, including the ridge, upper slope, middle slope, lower slope, valley, flatland) were taken as the independent variables and the tree height as the target variable, and a double hidden-layer BP neural network and a nonlinear mixed-effects model were established for tree height prediction using the MatLab 2016b software (MathWorks, Natick, MA, USA).

In this study, DBH and stand basal area were both used as input factors. DBH is the most crucial tree measurement indicator for individual trees, directly reflecting the growth status of the tree. It is a fundamental variable for constructing growth models and estimating biomass. The stand basal area of the forest represents the overall spatial occupancy level and competition intensity of the forest, reflecting the characteristics of the population structure. The simultaneous use of forest stand basal area and DBH as input factors for the model has theoretical rationality and practical feasibility, as supported by relevant research [21]. The two correspond to individual scale and forest scale respectively, with complementary information dimensions and no complete linear redundancy. DBH reflects the growth status of individual trees, while stand basal area reflects the overall and density competition of the forest stand. Therefore, it is reasonable to input them as independent variables into the model. Introducing models that can simultaneously capture individual differences and group competition effects in forest growth, site quality evaluation, carbon storage estimation, etc., significantly improves fitting accuracy and explanatory power. In forest growth simulation, yield estimation, and intelligent monitoring models, the joint input of DBH and stand basal area is a scientifically feasible optimal solution.

The dataset covers the sample plots distribution across six slope position categories (ridge: 12.79%; upper slope: 15.12%; middle slope: 22.09%; lower slope: 18.60%; valley: 16.28%; flatland: 15.12%). Descriptive statistics (e.g., mean, standard deviation) were calculated for all categories. The results show that the sample sizes across categories are relatively balanced, and there are certain differences in the distribution of tree height, DBH, and stand basal area. This supports the rationality of incorporating slope position as a random effect into the model.

Introducing slope position as a random effect into tree height growth models can significantly improve model fitting and prediction robustness. Slope position influences the redistribution of water and nutrients on the slope surface, as well as micro-environmental conditions such as light and temperature, thereby creating differentiated habitats like upper, middle, and lower slopes. This directly affects tree root distribution, nutrient uptake, and photosynthetic efficiency, leading to obvious spatial variations in tree height growth. By setting slope position as a random effect, it not only quantitatively captures topographic heterogeneity unexplained by fixed factors, reducing model residuals and heteroscedasticity, but also avoids loss of sample degrees of freedom due to the introduction of excessive categorical variables, making it particularly suitable for small-sample modeling. From an ecological perspective, the random effect of slope position reflects the regulatory role of micro-habitat heterogeneity driven by topography on tree growth and demonstrates the potential constraints of site conditions on tree height growth, enhancing the model’s alignment with the actual physiological and ecological processes of forest growth.

### 2.2. BP Neural Network

A BP neural network is a machine learning method utilizing a multi-layer feed-forward network trained by a BP error algorithm, usually composed of input, hidden, and output layers [22]. The structural parameters of this network include the number of hidden layers, the number of nodes in each hidden layer, and the transfer function between different layers. According to Kolmogorov’s theory [16], a single hidden-layer can satisfy the requirement of most general problems; however, for a complex system, two or more hidden layers may achieve better results [23]. Thus, the target of our study was optimizing the number of hidden-layer neurons in the network.

#### 2.2.1. Number of Hidden-Layer Nodes and Transfer Function

The calculation of the number of nodes in each hidden layer is an important issue in the field of neural networks. This problem is uncertain and the optimal number depends on various factors such as data size, activation function, network structure, etc. [24]. Since the 1990s until now, researchers have conducted a series of studies on this topic [25,26,27,28]. Among the many available solutions, Formula (1) [25,29] is chosen to calculate the number of nodes in the hidden layer.

(1) $$C=\sqrt{g+o}+r$$

where C is the number of hidden-layer nodes; g, the number of input-layer nodes; o, the number of output-layer nodes; and r, any integer between 1 and 10.

For the transfer function between the input and the hidden layers, or between the adjacent hidden layers includes tansig and logsig, the corresponding expressions are shown as Equations (2) and (3). The transfer function between the hidden and the output layers is purelin, as expressed in Equation (4):

(2) $$\tan sig(x)=\frac{2}{1+e^{-2x}}-1$$

(3) $$\log sig(x)=\frac{1}{1+e^{-x}}-1$$

(4) purelin(x)=ax+b

#### 2.2.2. Normalization and Denormalization of Input/Output Data

The map-min-max function was used to normalize the input and output data, mapping to [−1, 1]. The normalization procedure is expressed as Equation (5):

(5) $$y=\frac{\left(y_{\max}-y_{\min}\right)\times \left(x-x_{\min}\right)}{\left(x_{\max}-x_{\min}\right)}+y_{\min}$$

where y represents the normalized value, and the maximum value $y_{\max}=1$ and the minimum value $y_{\min}=-1$; x is the input/output value to be normalized, the maximum and minimum values of which are $x_{\max}$ and $x_{\min}$ respectively.

Denormalization can map the normalized value from [−1, 1] to the actual value.

#### 2.2.3. Model Training

The target of this phase is to minimize the loss function, which is used to measure the difference between the predicted and the observed value [30]. The commonly used loss functions include absolute value loss function, squared error loss function, Huber loss function, Logarithmic hyperbolic cosine (log-cosh) loss function, and Quantile loss function [31]. In terms of usability and efficiency, the squared error loss function was selected.

Newton’s method is an iterative solution method to minimize the loss function, which is based on the second-order Taylor series [32,33,34]. Compared with other optimization methods suitable for neural network training, such as the gradient descent method [35], conjugate gradient method [36], and heuristic method (simulated annealing algorithm, genetic algorithm, etc.) [37], Newton’s method has the advantages of fast convergence and good fitting, the basic formula for which is as follows:

(6) $$X\left(k+1\right)=X\left(k\right)-F^{-1}\left(k\right)\cdot J\left(k\right)$$

where $X\left(k+1\right)$ and $X\left(k\right)$ are the parameter values in the (k+1)-th and the k-th iterations respectively, and $F\left(k\right)$ and $J\left(k\right)$ are the second partial derivative (Hessian matrix) and the first partial derivative (Jacobian matrix) of $X\left(k\right)$ respectively.

The Hessian matrix of Newton’s method is composed of the second partial derivatives, which results in the large calculation load. In some cases, the Newton’s method may be failed if the Hessian matrix could not be positive definite.

In this case, Quasi-Newton method is proposed, which is based on the following “Quasi-Newton equation” [38]:

(7) $$l\left(k\right)=F\left(k\right)\times z\left(k\right)$$

(8) $$F^{-1}\left(k\right)\times l\left(k\right)=z\left(k\right)$$

The above two formulas are equivalent to each other, where $l\left(k\right)=J\left(k+1\right)-J\left(k\right)$, $z\left(k\right)=X\left(k+1\right)-X\left(k\right)$.

On the basis of Formulas (7) and (8), the approximate Hessian matrix with positive definite symmetry is constructed with iteration but without the calculation of second partial derivative, so as to reduce the significant computation of the Hessian matrix in Newton’s method.

The commonly used Quasi-Newtonian methods include DFP [39], BFGS [40], and L-BFGS [41]. DFP and BFGS can be used to calculate $F^{-1}\left(k\right)$ and $F\left(k\right)$ iteratively according to Formulas (8) and (9), respectively. L-BFGS reduces the space complexity of BFGS from $O\left(N^{2}\right)$ to $O\left(tN\right)$, where N is the order of matrix $F\left(k\right)$, and t is the parameter far less than N. Through analysis and testing carefully, the L-BFGS method was selected to minimize the loss function.

In addition to the loss function and optimization method, other parameters were set as follows: learning rate, 0.01; maximum iterations, 1000; target precision, 0.001; maximum number of verification failures, 20; and minimum performance gradient, 0.000001.

#### 2.2.4. Model Selection

As described in Section 2.2, a series of BP neural networks was obtained by setting the different structural parameters, such as the number of hidden layers and the transfer function. Then, the most suitable model was selected with the K-fold cross-validation [42].

Suppose the sample set S contains d samples, and the candidate models are $M_{1}$, $M_{2}$, …, $M_{h}$, then the process of K-fold cross-validation is as follows:

Step 1: The sample set S is randomly divided into K disjoint subsets, and the number of samples in each subset is d/K. These subsets are denoted as $S_{1}$, $S_{2}$, …, $S_{K}$.

Step 2: The following operation is carried out for every model $M_{e}$, e=1,2,…,h:

For n = 1 to K

Take the dataset $DS_{n}=S_{1}\cup \ldots \cup S_{n-1}\cup S_{n+1}\cup \ldots \cup S_{K}$ as the training set.

Train the model $M_{e}$ with the training set $DS_{n}$, so as to get the corresponding fitting function $H_{e,n}$.

Take the dataset $S_{n}$ as the validation set, then calculate the generalization error $\varepsilon_{S_{n}}\left(H_{e,n}\right)$ of $H_{e,n}$.

Generate the mean generalization error of model $M_{e}$ by calculating the average of the $\varepsilon_{S_{n}}\left(H_{e,n}\right)$, n=1,2,…,K.

Step 3: Choose the model $M_{opt}$ with the minimal mean generalization error as the optimal model.

Generally speaking, the mean square error (MSE) is used to represent the generalization error $\varepsilon_{S_{n}}\left(H_{e,n}\right)$, as shown in Equation (9).

(9) $$MSE=\frac{1}{w}\sum_{l=1}^{w}{\left(Y_{l}-{\hat{Y}}_{l}\right)}^{2}$$

where w is the number of samples, and Y and ${\hat{Y}}_{l}$ are the observed and the predicted values of the l-th samples respectively.

The “trial and error method” [43] may not traverse all the models corresponding to all the parameter combinations because its search step-size is often larger than 1. Therefore, it is necessary to carry out the second optimization based on the first optimization. In practice, in addition to the generalization error, the number of iterations, running time, and other factors are sometimes taken as the evaluation indicators.

#### 2.2.5. Neural Network Modeling Process

The process of tree height prediction modeling was as follows: firstly, the value range of the number of hidden layer nodes, transfer function, and other parameters were initially determined according to the data characteristics and modeling requirement; secondly, the appropriate step-size and value of the above parameters were accurately calculated with the “trial-and-error method” [43], and a series of candidate models was generated subsequently; thirdly, the optimal model was selected with K-fold cross-validation; in the end, the final model was produced by adjusting parameters around one of the previous optimal models with a step-size of 1.

The neural network model was generated according to the following parameters. The neurons of input-layer were DBH, stand basal area, and slope position, and the one in the output-layer was tree height. The transfer function was selected from logsig and tansig. There were two hidden layers, the number of whose neurons was from 3 to 12. The step-size of the “trial-and-error method” was 3, so the candidate numbers of neurons in the hidden-layer were 3, 6, 9, and 12. The MSE and the number of iterations were taken as the evaluation indicators whose priorities were first and second respectively.

### 2.3. Mixed-Effects Modeling

The mixed-effects model adopts the stand basal area and the *DBH* as the independent variables, the slope position as the random effect, and the tree height as the target variable. This model has integrated the mixed-effects and the covariance structure in order to describe the tree height and variability at the different slope positions.

#### 2.3.1. Mixed-Effects Model

The mixed-effects model first appeared in the 1970s [44], and was officially proposed by Laird et al. in 1981 [45], whose parameters partially or fully consist of two parts, i.e., fixed and random effects. According to the mixing form between mixed-effects and model, it can be divided into linear and nonlinear mixed-effects models. The model with linear relationship between them is called the linear mixed-effects model; others are known as the nonlinear one. Tree height is affected nonlinearly by many factors, and thus the nonlinear mixed-effects model can better describe tree height.

**Figure 1:**
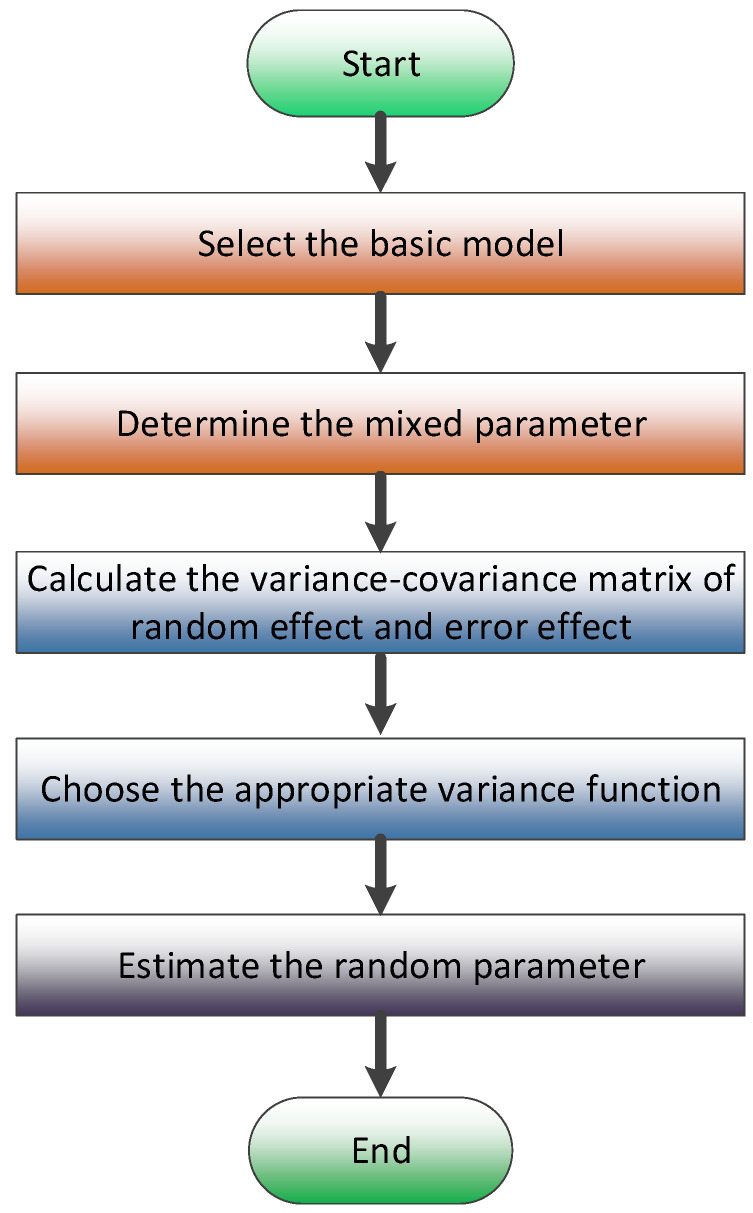
Flowchart for constructing the mixed model.

A mixed-effects model can be considered as the extension of a conventional model, which adds the random effect into the fixed parameters. For applying a mixed-effects model, the basic process is outlined in Figure 1. First of all, the basic model is selected; then, the mixed parameters to which the random effect will be added are determined. Next, the variance–covariance matrix of the random effect and the error effect are calculated successively. After that, the appropriate variance function is chosen to eliminate the heteroscedasticity problem that the error variance is increasing with the increase in the independent variable and results in the failure of the traditional independent equal-variance assumption. Finally, the random parameters of this model are estimated.

#### 2.3.2. Basic Model

Among the factors affecting the tree height, in addition to DBH, stand basal area and slope position are also included. In this study, DBH and stand basal area were considered as the fixed effects, slope position as the random effect, and tree height as the target variable, so as to construct the mixed-effects model. Using random effects to handle spatial heterogeneity and data non-independence issues can effectively improve the fitting accuracy and prediction reliability of tree height growth models.

After thorough analysis and comparison, the basic formula of this model [46,47] is expressed as Equation (10):

(10) $$H=1.3+\left(a+b\cdot BA\right)\cdot DG^{c}$$

where H represents the tree height; BA, the stand basal area; DG, the DBH; and a, b, and c, the parameters to be estimated.

We calculated the correlation coefficients between fixed effects and random effects, and the results showed that the correlation coefficient between slope position and cross-sectional area was −0.1396, and the correlation coefficient between slope position and DG was −0.0813, indicating that they can be used relatively independently for model construction. This study considered slope position as a random variable to investigate its impact on tree height.

The random effect slope position can be incorporated into different parameters of the model (e.g., a, b, and c). In the mixed-effects model of tree height growth, random effects can be introduced to single or multiple parameters such as asymptotic height, growth rate, and shape parameters to reflect differences among plots and across slope positions, thereby improving fitting and prediction accuracy.

#### 2.3.3. Structure of Mixed-Effects Model

The nonlinear mixed-effects model of tree height reflects the nonlinear dependence of the regression function on the fixed effects (DBH, stand basal area) and the random effects (slope position). The formula is expressed as Equation (11):

(11) $$H_{i,j}=f\left(\phi_{i},d_{i,j}\right)+\varepsilon_{i,j}$$

where i=1,2,…,m, m represents the number of slope positions; $j=1,2,\ldots ,n_{i}$, $n_{i}$, the number of measurements at the grade i slope position; and $\phi_{i}$, the parameter matrix of the grade i slope position. The size of $\phi_{i}$ is s×1; s is the number of parameters in this model, where s=3; $d_{i,j}=\left[DG_{i,j},BA_{i,j}\right]$, $DG_{i,j}$, and $BA_{i,j}$ are DBH and stand basal area derived from the *j*-th measurement on the grade i slope position. $\varepsilon_{i,j}$ is the prediction error; f, the nonlinear function representing the relationship between tree height and the fixed and the random effects; and $H_{i,j}$, the predicted value of tree height at the j-th measurement on the grade i slope position.

The parameter matrix $\phi_{i}$ reflects the change in *Larix olgensis* at the different slope positions, which is shown as Equation (12):

(12) $$\phi_{i}=A_{i}\beta +B_{i}z_{i},\;z_{i}∼N\left(0,D\right)$$

where β denotes the fixed effect parameter matrix with a size of p×1; and $z_{i}$ is the random effect parameter matrix with a size of q×1, which follows the normal distribution with a mean of 0 and variance–covariance matrix of D. p and q represent the number of the fixed and the random effects parameters respectively in this model, where p=2 and q=2; D, the variance–covariance matrix of random effect (slope position), reflecting the variation and difference between the different slope positions; $A_{i}$ and $B_{i}$, the designed matrixes of the fixed and the random effects at the grade i slope position.

#### 2.3.4. Calculating Parameter of Mixed-Effects Model

**Figure 2:**
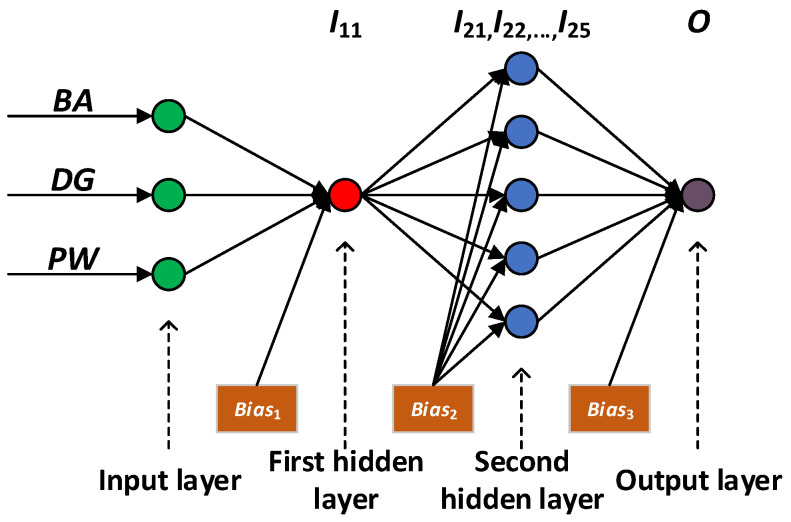
Tree height model based on the BP neural network.

After determining the structure of the mixed-effects model, the relevant parameters were calculated according to the procedure in Figure 2.

**(1)**  **Determining Parameter**

It is necessary to determine which parameter in Equation (11) should be added with the random effect before defining the covariance structure of the different slope positions. Thus, the parameters *a*, *b*, and *c* of this formula need to be tested with the appropriate criterion, such as AIC (Akaike Information Criterion), BIC (Bayesian Information Criterion), LL (Log Likelihood), and LLR (Log Likelihood Ratio). For the first three criteria, the smaller is better, but it is the opposite for the last criterion. It is found that adding the random effect (slope position) into the parameters a and b in Equation (11) had the better result.

**(2)**  **Calculating Variance–Covariance Matrix of Random Effect**

The variance–covariance matrix of the random effect represents the variability of slope position, which is all the same for every slope position. This matrix appeared as D in Section 2.3.3. Since the random effect was added into the parameters *a* and *b*, the size of D was 2×2, which is shown as follows:

(13) $$D=\left[\begin{array}{cc} \sigma_{u}^{2} & \sigma_{uv}^{2}\\ \sigma_{uv}^{2} & \sigma_{v}^{2} \end{array}\right]$$

where u and v represent the random effect parameters added into the parameters *a* and *b*; $\sigma_{u}^{2}$ and $\sigma_{v}^{2}$, the variances of u and v, respectively; and $\sigma_{uv}^{2}$, the covariance of the two parameters.

**(3)**  **Calculating Variance–Covariance Matrix of Error Effect**

The variance–covariance matrix of the error effect represents the error of slope position, which is different for every slope position. $R_{i}$ is one of the grade i slope positions, which is expressed as Equation (14).

(14) $$R_{i}=\sigma^{2}G_{i}^{0.5}E_{i}G_{i}^{0.5}$$

where $\sigma^{2}$ is the error’s variance, which is determined by the residual variance of this model; $G_{i}$ and $E_{i}$, with the same size of $n_{i}\times n_{i}$, are used to explain the heteroscedasticity and error’s autocorrelation of the grade i slope position respectively; $G_{i}$ is the diagonal matrix. Since every sample area only has one value in the dataset, there is no autocorrelation of multi-values from the same sample area, and thereby $E_{i}$ is the unit matrix.

In order to eliminate the heteroscedasticity, the following functions were considered: exponential function $\mathrm{var}\left(\varepsilon \right)=\sigma^{2}\exp\left(\alpha *DG\right)$, power function $\mathrm{var}\left(\varepsilon \right)=\sigma^{2}DG^{\alpha }$, and power function with constant $\mathrm{var}\left(\varepsilon \right)=\sigma^{2}{\left(\alpha_{1}+DG^{\alpha_{2}}\right)}^{2}$. Among them, α, $\alpha_{1}$, and $\alpha_{2}$ are parameters to be estimated, and DG is the DBH. The optimal residual variance model was selected from the above functions, which included smaller AIC and BIC, but bigger Log Likelihood (LL) was also possible.

**(4)**  **Estimating Random Parameter**

The random effect parameter matrix of the grade i slope position is estimated according to Equation (15).

(15) $$z_{i}=DL_{i}^{T}{\left(R_{i}+L_{i}DL_{i}^{T}\right)}^{-1}\varepsilon_{i}$$

where D and $R_{i}$ are variance–covariance matrixes as in Equations (14) and (15) respectively; $L_{i}$, the matrix with a size of $n_{i}\times q$, representing the partial derivatives of different predicted values with respect to random effect parameters at the grade i slope position; $L_{i}^{T}$, the transpose of $L_{i}$; and $\varepsilon_{i}$, the residual vector composed of the errors at the $n_{i}$ measurements of the grade i slope position.

### 2.4. Evaluation Index of the Two Models

Generally speaking, the coefficient of determination ($R^{2}$) is used to characterize the fitting quality through the change in the data. The closer the value is to 1, the stronger the model’s ability is to explain the dependent variable, and the model fits the data better. The smaller the root mean square error (RMSE) and the average absolute error (MAE), the higher the accuracy of the model fitting.

Thus, the $R^{2}$, RMSE, and MAE were used as the evaluation indices of the tree height model, as shown in Equation (16), Equation (17), and Equation (18), respectively:

(16) $$R^{2}=1-\sum_{l=1}^{w}\frac{{\left(Y_{l}-{\hat{Y}}_{l}\right)}^{2}}{{\left(Y_{l}-\bar{Y}\right)}^{2}}$$

(17) $$RMSE=\sqrt{\frac{1}{w}\sum_{l=1}^{w}{\left(Y_{l}-{\hat{Y}}_{l}\right)}^{2}}$$

(18) $$MAE=\frac{\sum_{l=1}^{w}\left|Y_{l}-{\hat{Y}}_{l}\right|}{w}$$

In Equation (16), Equation (17), and Equation (18), w is the number of samples; $\overset{-}{Y}$ is the mean measurements of the samples; and $Y_{l}$ and ${\overset{\wedge }{Y}}_{l}$ are the measurement and predicted values of the l-th sample, respectively.

## 3. Results

### 3.1. Data Characteristic

The statistical characteristics of the above data are summarized in Table 1.

### 3.2. Strucutre of Neural Nework

A series of candidate models was generated according to the method in Section 2.2.4, which are shown in Table 2.

It can be seen that the MSE reached the minimum of 0.00136 when the neuron distribution was “3:3:6:1” and the transfer functions were both “tansig”. The corresponding number of iterations was 30.4, which was also less than the one in the most other situations. Thus, this candidate model was taken as the optimal model according to the K-fold cross-validation in Section 2.2.4, where K = 5.

For the further optimization, the number of neurons in the hidden-layer of the optimal model was adjusted with a step-size of 1. Thus, its testing range in the first and the second hidden layers was “1, 2, 3, 4, 5” and “4, 5, 6, 7, 8” respectively. The corresponding training results are listed in Table 3.

It can be seen that when the neurons distribution was “3:1:5:1”, the MSE obtained the minimum of 0.0133, which was smaller than the one of 0.0136 in Table 2. The final model generated by this parameter had the highest precision. The number of iterations in that network was 30, which was not the minimum. But it was obviously less than the average iterations of 33.475 in Table 3, which fully shows its advantage of speed.

The final neural network is shown in Figure 2. The BA, DG, and PW of the input layers represent the stand basal area, DBH, and slope position respectively. The neuron in the first hidden-layer is $I_{1,1}$; those in the second hidden-layer are $I_{2,1}$, $I_{2,2}$, $I_{2,3}$, $I_{2,4}$, and $I_{2,5}$. The biases between the input-layer and the first hidden-layer, the first and the second hidden layers, and the second hidden-layer and the output-layer were Bias_1_, Bias_2_, and Bias_3_, respectively, as follows:

$$Bias_{1}=\left[2.3042\right]$$

$$Bias_{2}=\left[7.0795,\;3.3531,\;0.1008,\;4.4650,\;7.0193\right]$$

$$Bias_{3}=\left[0.4914\right]$$

In Figure 2, the transfer function from the input-layer to the first hidden-layer, as well as that from the first to the second hidden-layer, was tansig, whereas, the one from the second hidden-layer to the output-layer was purelin. According to the weight matrix and the corresponding bias, the transfer function from the input-layer to the first hidden-layer is as follows:

$$I_{1,1}=\tan sig\left(0.0170*BA+0.1813*DG+0.0015*PW+2.3042\right)$$

The transfer functions from the first to the second hidden-layer are as follows:

$$I_{2,1}=tansig\left(-7.1615*I_{1,1}+7.0795\right)$$

$$I_{2,2}=tansig\left(-7.1550*I_{1,1}+3.3531\right)$$

$$I_{2,3}=tansig\left(6.9874*I_{1,1}+0.1008\right)$$

$$I_{2,4}=tansig\left(6.5161*I_{1,1}+4.4650\right)$$

$$I_{2,5}=tansig\left(6.9861*I_{1,1}+7.0193\right)$$

The transfer function from the second hidden-layer to the output-layer is as follows:

$$O=\mathrm{purelin}\left(-16.9476*I_{2,1}+1.0247*I_{2,2}-1.2429\;I_{2,3}+0.8921I_{2,4}+2.0557I_{2,5}+0.4914\right)$$

### 3.3. Construction of Mixed-Effects Model

The random effect (slope position) was added into the parameters a and b in Equation (11). The heteroscedasticity was eliminated according to the procedure in the section “Calculating Variance–Covariance Matrix of Error Effect”, the result of which is shown in Table 4.

It can be seen that the result of the exponential function had the better AIC, BIC, and LL values than the other two functions. Therefore, the exponential function was selected to eliminate the heteroscedasticity.

All data were divided into training set (64 samples) and test set (22 samples), so as to establish the mixed-effects model, the parameters of which are listed in Table 5.

By using the results in Table 4 and Table 5, the formula for the tree height prediction of *Larix olgensis* based on the nonlinear mixed-effects model was finally determined as follows:

(19) $$H_{i,j}=1.3+\left[\left(1.2306873+u_{i}\right)+\left(0.0034150+v_{i}\right)\cdot BA_{i,j}\right]\cdot DG_{i,j}^{0.8759386}+\varepsilon_{i,j}$$

where $BA_{i,j}$*,* $DG_{i,j}$*,* $H_{i,j}$, and $\varepsilon_{i,j}$, respectively, are the stand basal area, DBH, predicted value, and error at the j-th measurement on the grade i slope position as in Equation (12); $u_{i}$ and $v_{i}$ are the random effect parameters of the grade i slope position, which follows the normal distribution with a mean of 0 and variance–covariance matrix of D, as shown in Equation (20):

(20) $$\left[\begin{array}{c} u_{i}\\ v_{i} \end{array}\right]\sim N\left(0,D\right)\;D=\left[\begin{array}{cc} 0.00000460513 & -0.00000122332\\ -0.00000122332 & 0.00000044662 \end{array}\right]$$

$\varepsilon_{i,j}$ also follows the similar normal distribution as shown in Equation (21):

(21) $$\varepsilon_{i,j}\sim N\left(0,R_{i}\right)\;R_{i}=1.234501^{2}\cdot G_{i}^{0.5}I_{i}G_{i}^{0.5}$$

where $R_{i}$, $G_{i}$, and $I_{i}$ are as described in Equation (15); the number of diagonal elements of $G_{i}$ is equal to the number of measurements at the grade i slope position. The j-th diagonal element ${\left[G_{i}\right]}_{j}$ is calculated as follows:

(22) $${\left[G_{i}\right]}_{j}=\exp\left(-0.04903804\cdot DG_{i,j}\right)$$

Then, the formula of $\varepsilon_{i,j}$ can be expressed synthetically as follows:

$$\mathrm{var}\left(\varepsilon_{i,j}\right)=1.234501^{2}\exp\left(-0.04903804\cdot DG_{i,j}\right)$$

Thus, the tree height of *Larix olgensis* was predicted by substituting the measurements $BA_{i,j}$ and $DG_{i,j}$ *and* parameters $u_{i}$, $v_{i}$, and $\varepsilon_{i,j}$_i_ into Equations (19)~(22).

### 3.4. Comparsion Results of Evaluation Index

In this study, the DBH, the stand basal area, and the slope position were used as the input factors, and the tree height was used as the output factor. The tree height model was established by using a double hidden-layer neural network. Concurrently, the slope position was taken as the random effects, whereby the mixed-effects model was used to establish the tree height model. In order to compare the effects of the above two methods, precision analysis was performed.

The numbers of samples in training set and test set were 64 and 22. The neural network and the mixed-effects models were compared by using the evaluation indices defined by Equations (16)–(18). The results are listed in Table 6.

In Table 6, the *R*^2^ of the double hidden-layer BP neural network was 0.9068, which is higher than the mixed-effects model. That is to say, the neural network accounted for about 90.68% of the tree height variation, but the mixed-effects model only 85.9%. The RMSE and MAE in the neural network were 18.69% and 43.79% lower than the mixed-effects model, respectively. It can be observed from the overall levels that the double hidden-layer BP neural network is superior to the nonlinear mixed-effects model in tree height prediction. Although the coefficient of determination (*R*^2^) for the tree height model increased by 4%, tree height is the basis for calculating forest stock and carbon sinks. Under the current trend of precision forestry, precise tree height plays an important role in calculating forest stock and carbon sinks, and has significant meaning and value for forest management and other aspects.

From Table 3, it can be seen that the best neural network “3:1:5:1” has an RMSE of 0.03688 in the training set (derived from the mean square error in this table: 0.00136). From Table 6, it is known that this network has an RMSE of 1.3197 in the validation set. By comparison, it can be concluded that this network has overfitting, and the reason is that the size of the dataset is relatively small (86 records). However, judging the model’s performance solely based on RMSE is not comprehensive; it is necessary to conduct a comparative analysis through the fitting of “predicted value—observed value” and the residual distribution.

**Figure 3:**
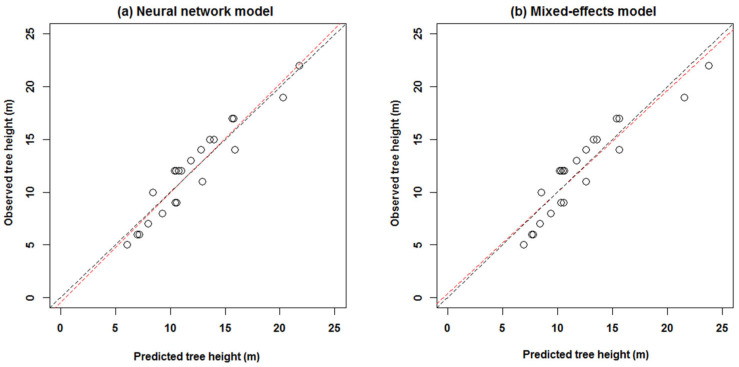
Comparison between predicted value and measured value of two tree height models.

The predicted and measured values of the two tree height models were compared, the results of which are plotted in Figure 3.

In this figure, the circles represent the sample points, and the red dotted lines represent the fitting equations. The left subgraph is the scatter diagram of neural network model, and the right subgraph is the one of mixed-effects model. The fitting equations for the two models are *y* = 1.0384*x* − 0.4265 and *y* = 0.9637*x* + 0.3631 respectively, where *x* and *y* are the predicted and measured values respectively. If the fitting equation is closer to *y* = *x*, the predicted value is closer to the measured value, which indicates that the corresponding prediction model is better.

**Figure 4:**
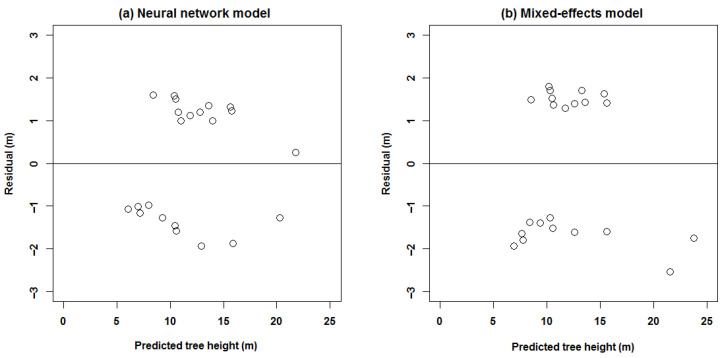
Residual distribution based on BP neural network and mixed-effects models.

The residual is the difference between the predicted and the measured values. The more concentration around 0, the higher the prediction accuracy. The residual distribution of the predicted values of the two tree height models is plotted in Figure 4.

In this figure, the circles represent the sample points. It can be observed in Figure 4 that within the double hidden-layer BP neural network, there were two plots in a residual interval of (−1, 1), accounting for 9.09%; the residual interval of (−2, 2) had 22 plots, accounting for 100%. When using the mixed-effects model, there were zero plots in a residual interval of (−1, 1) and 21 plots in a residual interval of (−2, 2), accounting for 95.45%. It can also be observed that the difference between the predicted value and observed value of the double hidden-layer BP neural network was significantly lower than that of the nonlinear mixed-effects model.

From the above results, the fitting effect of the neural network model is slightly better than that of the mixed model, and their residual distributions conform to the model assumptions. Thus, it can be concluded that through the reasonable effect setting and model selection, the overfitting risk has been successfully controlled, and the constructed model has a good fitting effect.

In summary, the double hidden-layer BP neural network is superior to the nonlinear mixed-effects model in tree height prediction.

## 4. Discussion

We established two kinds of models. One is the double hidden layer BP neural network, and the other is the mixed-effects model. Among these models, the best double hidden-layer BP neural network was determined according to MSE and iterations, which is used as the optimal height–diameter model for *Larix olgensis* forest plantation in Jilin Province, China. *Larix olgensis* is one of the dominant fast-growing afforestation tree species, which is important in wood production and other aspects and is widely used in the electricity industry, coal mines, manufacturing ships, bridges, railways, etc. Thus, *Larix olgensis* was taken as the research objective in our study. This study compared the prediction effects of double-layered BP neural network and mixed-effects models. The experimental results indicated that the higher performance was obtained with two hidden layers, which includes higher fitting precision, higher estimating efficiency, and fewer iterations [23]. That is to say, the double hidden-layer BP neural network appears substantially superior to the mixed-effects model in prediction of tree height (Table 6; Figure 3 and Figure 4). Our results are not consistent with those from Castaño-Santamaría et al. [12] and Özçelik et al. [13], which predict tree height of the uneven-aged beech forests in northwestern Spain and Crimean juniper in the southwestern region of Turkey. They compared the neural network model against the mixed-effects model and found the latter was better. I do not think this discrepancy comes from tree species used. The main reason is that we use the double hidden layer neural network, but they only apply a single hidden-layer neural network. For example, Özçelik et al. only used the single hidden-layer with only one or two nodes, and did not investigate the effects of multiple hidden layers on the precision and determine the appropriate transfer functions. In addition, the “trial and error approach”, k-fold cross-validation, and combinatorial optimization [23] were adopted to select the optimal model, which is substantially different from the previous studies [8,10,12,13]. It can help to optimize the structure of the neural network, such as the transfer functions and the number of hidden layers and its nodes, which has substantial effects on the precision of the neural network model. Castaño-Santamaría [12] considered the change in input factors, but did not take into account other factors, such as different transfer functions, and did not introduce the process of determining the optimal neural network. Castro et al. [48] established the multi-layer perceptron neural network growth model for Eucalyptus. They estimated the annual mortality of the best structure with three, four, and one neurons in the input, hidden, and output layers respectively. All these studies [12,48] compared the precisions using different input variables, but did not compare the precisions using different transfer functions and the number of neurons. In our study, the neural network modeling produced the higher fitting precision compared to the mixed-effects models (Table 6). Generally speaking, the neural network has the strong robustness, and the mixed-effects model has biological significance, such as the parameters describing growth rate and pattern. But it is very complex to determine the optimal parameter of the mixed-effects model compared with the procedure of the neural network. Furthermore, the BP neural network model has better generalization ability, and therefore can approximate any nonlinear continuous function with high precision. The BP neural network generally follows nonlinear patterns, which make it suitable for solving the problems caused by factors affecting plant growth. The process of determining the traditional height–diameter model and neural network height–diameter model was also evaluated in our study. The traditional regression modeling needs the comparison and evaluation of the fitting precisions of the candidate models, and the model with the highest precision could be selected as the final model. The neural network model should determine the number of hidden layers and neurons in each layer, and the form of the transfer function according the precision and iterations, and the optimal model can be generated with the best structure.

Due to the small size of the dataset, the trained neural network exhibits overfitting to a certain extent. However, by comparing the “predicted value—observed value” fitting and residual distribution with the mixed-effects model, the fitting effect of the neural network model is slightly better than that of the mixed model, and the residual distribution also conforms to the model assumptions. This indicates that the constructed model can control the risk of overfitting and the prediction effect meets the actual needs.

In addition, this study is different from the authors’ previous study [23]. In previous research, the input factor of the neural network only considered the DBH. In addition to DBH, this research considered two additional factors, i.e., stand basal area and slope position. In the authors’ previous study [23], the input factor of the neural network and mixed-effects models was different. One used DBH, the other DBH and sample plot level. But the input factors in this study were both the same. That is to say, the result of this study is more convincing. In addition, the tree species studied was different. The tree species in the previous study was *Poplar*, while that in this study was *Larix olgensis*. But the research results are basically the same, which means that the fitting accuracy of the neural network is higher than that of the mixed-effects model.

## 5. Conclusions

Improvement of tree height prediction accuracy is not only a necessity for forestry production, but is also key to laying a solid foundation for carbon reduction research and quantifying the ecological value of forests. Deepening the optimization of tree height prediction technology cannot only solve the pain points in forestry resource monitoring, but also provide strong support for national carbon cycle research and climate governance decisions. It has both theoretical innovation significance and practical application value, and is a core research direction in the forestry and ecological fields.

This study proposed a method for optimizing the hidden-layer number, node number, and transfer function of tree height prediction models based on the BP neural network. First, the “trial-and-error method” was used to determine the value of the aforementioned parameters, and several models were established. Then, by using K-fold cross-validation, the optimal neural network was initially screened according to the mean square error and the number of iterations. After that, the near-domain search was performed around the structure of the optimal neural network with a step-size of 1, and a number of models was generated. K-fold cross-validation was again implemented. Finally, the final neural network was determined using the evaluation indicators of mean square error and number of iterations for K-fold cross-validation.

The *Larix olgensis* plantation in Jilin Province was used to establish and optimize the tree height prediction model. The results indicated that the final neural network structure was 3:1:5:1, and the transfer functions from the input-layer to the first hidden-layer and from the first to the second hidden-layer were both tansig. Thus, this model accounted for about 90.68% of the tree height variation. On the basis of determining the basic model, by introducing random effects, calculating the variance–covariance matrix, and estimating random parameters, the mixed-effects model was established for comparison with the neural network model. By using R^2^, RMSE, and MAE as evaluation indicators, we compared the fitting precision of the two models in tree height prediction, as well as the residual distribution. The experimental results indicate that the double hidden-layer BP neural network is superior to the nonlinear mixed-effects model in tree height prediction, although it takes a lot of time in model construction (setting number of layers and nodes, and transfer functions) and model training.

In this study, the slope position was regarded as a random effect in the prediction of tree height. This approach can control the spatial heterogeneity and intra-group correlation of the site, save degrees of freedom, improve the efficiency of model prediction, and distinguish between fixed driving factors and random site fluctuations. The experimental results show that this method can suppress overfitting and improve the prediction accuracy. Currently, there are relatively few studies in this area, which is of certain forward-looking and innovative significance.

To sum up the above statement, this paper has compared the tree height prediction models of a double hidden-layer BP neural network and a nonlinear mixed-effects model, which is useful for selecting the appropriate method in tree height prediction. In addition to the slope position, the stand mean height is also affected by other factors. In subsequent studies, these factors need to be incorporated into the tree height prediction model so as to improve the prediction accuracy.

## Figures and Tables

**Table 1:** Statistical characteristics of the *Larix olgensis* data.

Characteristic	Min	Max	Mean	Standard Deviation
DBH/cm	5.7	26.1	12.4	4.3
Stand basal area/m^2^·hm^−2^	1.35	32.56	15.44	7.97
Tree height/m	5	22	12.8	3.8

**Table 2:** Training results from neural networks.

Neurons in Each Layer	MSE	Iterations	MSE	Iterations	MSE	Iterations	MSE	Iterations
	log:log		tan:tan		log:tan		tan:log	
3:3:3:1	0.0613	35.0	0.0242	35.6	0.0280	53.2	0.0295	42.8
3:3:6:1	0.0278	35.0	**0.0136**	**30.4**	0.0210	42.2	0.0234	34.0
3:3:9:1	0.0146	31.4	0.0188	40.8	0.0416	37.2	0.0163	30.0
3:3:12:1	0.0244	29.8	0.0180	36.2	0.0357	31.0	0.0255	36.8
3:6:3:1	0.0262	30.6	0.0283	31.2	0.0310	29.6	0.0561	24.0
3:6:6:1	0.0304	35.6	0.0279	37.8	0.0360	22.0	0.0216	28.8
3:6:9:1	0.0243	46.8	0.0190	38.8	0.0368	29.0	0.0278	36.4
3:6:12:1	0.0232	38.0	0.0242	32.0	0.0642	22.4	0.0322	34.6
3:9:3:1	0.0545	40.8	0.0250	28.0	0.0412	24.4	0.0184	50.6
3:9:6:1	0.0362	29.0	0.0271	24.2	0.0265	31.2	0.1304	35.2
3:9:9:1	0.0357	39.0	0.0142	29.6	0.0385	24.8	0.0390	26.4
3:9:12:1	0.0360	29.2	0.0401	33.8	0.0631	48.8	0.0361	28.8
3:12:3:1	0.0310	41.4	0.0326	21.4	0.0423	35.6	0.0244	26.6
3:12:6:1	0.0553	26.2	0.0208	31.6	0.0356	37.8	0.0330	33.2
3:12:9:1	0.0402	34.2	0.0400	27.6	0.0211	41.8	0.0271	36.8
3:12:12:1	0.0200	34.2	0.0697	25.0	0.0205	32.8	0.0273	34.0

**Table 3:** Training results from local optimized neural networks.

Neurons in Each Layer	MSE	Iterations	Neurons in Each Layer	MSE	Iterations	Neurons in Each Layer	MSE	Iterations
	tan:tan			tan:tan			tan:tan	
3:1:4:1	0.0158	27.6	3:2:7:1	0.0838	37.8	3:4:6:1	0.0710	40.8
3:1:5:1	**0.0133**	**30.0**	3:2:8:1	0.0460	29.4	3:4:7:1	0.0266	43.6
3:1:6:1	0.0952	31.0	3:3:4:1	0.0164	39.2	3:4:8:1	0.0172	41.2
3:1:7:1	0.0624	28.0	3:3:5:1	0.0480	24.6	3:5:4:1	0.0191	35.2
3:1:8:1	0.0192	29.8	3:3:7:1	0.0549	31.6	3:5:5:1	0.0317	26.6
3:2:4:1	0.0166	41.4	3:3:8:1	0.0339	29.4	3:5:6:1	0.0200	31.4
3:2:5:1	0.0606	32.4	3:4:4:1	0.0265	28.2	3:5:7:1	0.0164	36.0
3:2:6:1	0.0620	28.0	3:4:5:1	0.0186	44.4	3:5:8:1	0.0177	35.8

**Table 4:** Residual simulation results from nonlinear mixed model for predicting height of *Larix olgensis*.

Residual Variance Model	Formula	AIC	BIC	LL
Exponential function	$\mathrm{var}\left(\varepsilon \right)=\sigma^{2}\exp\left(\alpha *DG\right)$	147.2112	164.4823	−65.60562
Power function	$\mathrm{var}\left(\varepsilon \right)=\sigma^{2}DG^{\alpha }$	147.927	165.1981	−65.9635
Power function with constant	$\mathrm{var}\left(\varepsilon \right)=\sigma^{2}{\left(\alpha_{1}+DG^{\alpha_{2}}\right)}^{2}$	149.927	169.357	−65.96352

**Table 5:** Parameters of mixed-effects model for predicting height of *Larix olgensis*.

Items	Name	Value	Standard Deviation	*p* Value
fixed parameters	a	1.2306873	0.09217312	*p* = 0.0000 < 0.05
	b	0.0034150	0.00158609	*p* = 0.0356 < 0.05
	c	0.8759386	0.03105736	*p* = 0.0000 < 0.05
variance	$\sigma_{u}^{2}$	0.00000460513		
	$\sigma_{v}^{2}$	0.00000044662		
	$\sigma_{uv}^{2}$	−0.00000122332		
	$\sigma^{2}$	1.2345006919		

**Table 6:** Accuracy comparison between BP neural network and mixed-effects models.

Models	R^2^	RMSE	MAE
Neural network model	0.9068	1.3197	1.2736
Mixed-effects model	0.8590	1.6230	2.2658

## Data Availability

Data used in this study are available from the National Forestry and Grassland Science Data Center (http://www.cfsdc.org/).
